# Inhaled antibiotic-loaded polymeric nanoparticles for the management of lower respiratory tract infections

**DOI:** 10.1039/d1na00205h

**Published:** 2021-05-17

**Authors:** Mohammad Zaidur Rahman Sabuj, Nazrul Islam

**Affiliations:** Pharmacy Discipline, School of Clinical Sciences, Queensland University of Technology (QUT) Brisbane QLD Australia nazrul.islam@qut.edu.au; Institute of Health and Biomedical Innovation (IHBI), Queensland University of Technology (QUT) Brisbane QLD Australia; Centre for Immunology and Infection Control (CIIC), Queensland University of Technology (QUT) Brisbane QLD Australia

## Abstract

Lower respiratory tract infections (LRTIs) are one of the leading causes of deaths in the world. Currently available treatment for this disease is with high doses of antibiotics which need to be administered frequently. Instead, pulmonary delivery of drugs has been considered as one of the most efficient routes of drug delivery to the targeted areas as it provides rapid onset of action, direct deposition of drugs into the lungs, and better therapeutic effects at low doses and is self-administrable by the patients. Thus, there is a need for scientists to design more convenient pulmonary drug delivery systems towards the innovation of a novel treatment system for LRTIs. Drug-encapsulating polymer nanoparticles have been investigated for lung delivery which could significantly reduce the limitations of the currently available treatment system for LRTIs. However, the selection of an appropriate polymer carrier for the drugs is a critical issue for the successful formulations of inhalable nanoparticles. In this review, the current understanding of LRTIs, management systems for this disease and their limitations, pulmonary drug delivery systems and the challenges of drug delivery through the pulmonary route are discussed. Drug-encapsulating polymer nanoparticles for lung delivery, antibiotics used in pulmonary delivery and drug encapsulation techniques have also been reviewed. A strong emphasis is placed on the impact of drug delivery into the infected lungs.

## Abstract 2

A schematic diagram of pulmonary drug delivery from the DPI formulation, which consists of drug encapsulated in polymer nanoparticles. Nanoparticles are aerosolized using a DPI device and the patient's inspiratory force for lung deposition.

## Graphical abstract

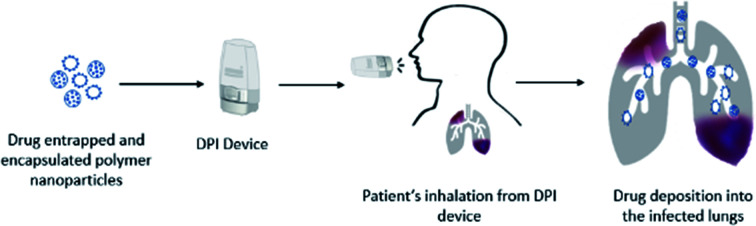

## 1. Introduction

Lower respiratory tract infections (LRTIs) are one of the major lung diseases caused by the pathogenic bacteria *Pseudomonas aeruginosa* and *Streptococcus pneumonia,* which are associated with cystic fibrosis (CF), chronic obstructive pulmonary disease (COPD) and bronchiectasis. Some other microorganisms are also considered as the causative agents of LRTIs including respiratory syncytial virus, fungus, and mycoplasmas. However, some environmental substances (tobacco smoke, dust, chemicals, vapours, allergens, and air pollution) can also cause inflammation and damage lung cells, which produce excessive mucus in the small air sacs and lead to an infection.^1^ The Global Burden of Diseases, Injuries and Risk Factors Study (GBD) defined LRTIs more precisely as pneumonia or bronchitis, which is one of the leading causes of morbidity and mortality worldwide.^2^*S. pneumonia*, the major causative agent of pneumonia, was listed as the leading cause of LRTIs, which contributed to more deaths than all other diseases.^3^ In addition, LRTIs were ranked as the sixth leading cause of death for all ages and the major cause of death among children younger than 5 years and contributed to the total recorded death of 2.38 million.^2^ Antibiotics are the first line treatments for LRTIs. In severe cases, patients need to be hospitalized where they are treated with oral or intravenous antibiotics.^1^ Currently available treatment systems require long term administration of drugs at high doses. Thus, the dose-dependent toxicity of drugs makes the currently available treatment system less efficient. Therefore, the establishment of a proper management system for LRTIs is of pivotal importance for researchers.

Pulmonary drug delivery technology is based on the delivery of inhalable formulations (micronized dry powders, solutions, or suspensions) which are aerosolized by suitable devices and deposited into the deep lungs. This route of drug delivery is efficient in delivering drugs directly into the deep lungs. Therefore, pulmonary drug delivery has several advantages over traditional systemic drug delivery methods, including rapid onset of drug action at a very low dose, reduced dose related adverse effects and improved patient compliance.

Pulmonary drug delivery for LRTIs using nanoparticles is one of the emerging strategies to fight against the antibiotic resistant microorganisms, especially *P. aeruginosa*.^4^ Nanoparticle based drug delivery includes several carriers in nano-size, such as polymeric micelles, drug polymer conjugates, liposomes, and dendrimers.^5^ The drug loaded polymer nanoparticles can provide several benefits for drug delivery systems including protecting the drug from degradation under unfavourable conditions, increasing drug solubility and absorption through epithelium by providing easy diffusion, preferential distribution of drugs within the target cells and improved therapeutic effects. In addition, polymer-based drug nanoparticle surfaces are modifiable, which improves the drug release pattern in a more controlled fashion; thus the desired therapeutic effects can be achieved for a long time.^6–8^

This review provides an overview on LRTIs, available treatments against this disease and their drawbacks. Then, a brief description on the pulmonary drug delivery system in light of the suitability to introduce it against LRTI treatment and the challenges of pulmonary drug delivery in the infected lungs are discussed. Polymer based inhalation delivery, excipients, and drug–polymer conjugation to form nanoparticles for pulmonary delivery are also described. Finally, common preparation techniques used to synthesize drug-encapsulating polymer nanoparticles for lung delivery are described.

## 2. An overview of the respiratory tract infections

Respiratory tract infection (RTI) is an infection within the lungs which affects both upper and lower respiratory tracts.^9^ More precisely it is regarded as any respiratory illness which refers to a variety of infections in the nose, sinuses, throat, airways and lungs. Most of the illness related to RTIs do not need medication and get better gradually.^10^ However, in some severe cases antibiotics are the only drugs used for the treatment. Rapid diagnosis is important to identify the causative agents and provide timely therapeutic intervention.^11^ Both viruses and bacteria can cause RTIs and in most cases they spread through direct contact, airborne particles and droplets from an infected person.^12^ Viral RTIs constitute a major public health issue because of their extensive incidence, ease of transmission and significant rate of morbidity and mortality.^13^ Children are two or three times more susceptible than adults with acute viral RTIs.^14^ Common upper respiratory tract infections (URTIs) include cold, sinusitis, tonsillitis and laryngitis, while LRTIs include influenza, pneumonia, bronchitis and bronchiolitis.^15^ Pneumonia, the common LRTI is caused by virus or bacteria and less commonly by fungi.^16^ The alveoli in the lungs fill with secretions and fluid, decreasing the ability for oxygen to be transported across the tissue to adequately oxygenate vital organs.^17^ Symptoms include shortness of breath and hypoxia and patients often need oxygen therapy and ventilation support. As such, pneumonia is considered the most severe case of LRTIs.^18^

## 3. Details on lower respiratory tract infections (LRTIs)

**Fig. 1:**
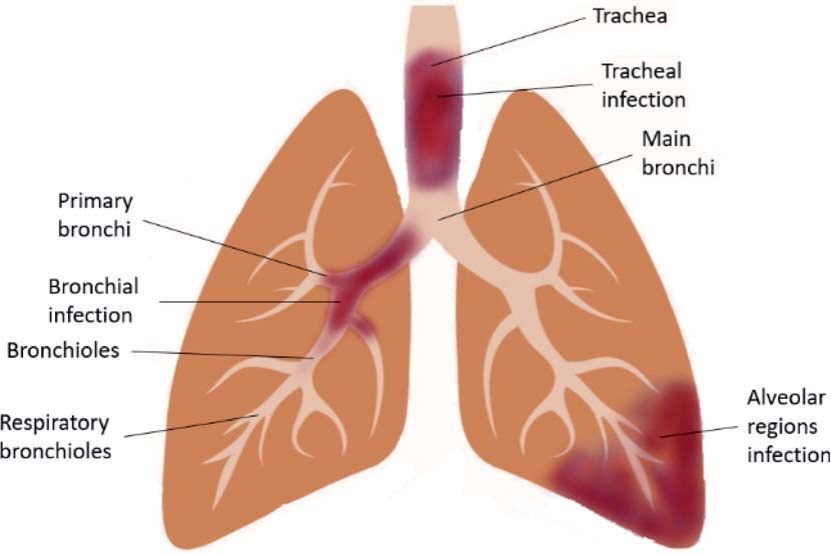
Schematic diagram of the infected lower respiratory tract.

LRTI (Fig. 1) is a fatal lung infection that lasts for up to several weeks and appropriate diagnosis and treatments are required, otherwise the serious infection causes the patient's death. It usually shows symptoms such as coughing and sputum production, palpitation, wheeze, chest pain and shortness of breath. Symptoms of LRTIs usually depend on the type of infection and its severity. It is often associated with other lung disorders such as COPD and CF.^19^ Both Gram-positive and Gram-negative bacteria are the causative agents of LRTIs, whereas viral infections are also found in COPD.^20^ Microorganisms that are responsible for typical bacterial infection include *Staphylococcus aureus*, *Klebsiella pneumonia*, and *Haemophilus influenza*; whereas, atypical bacterial infection is caused by *Chlamydophila pneumonia*, *Mycoplasma pneumonia*, *Legionella pneumonia* and *Chlamydia psittaci*.^21^ Accurate detection of the causative agents can be done using molecular diagnosis of the infected respiratory tract.^22^

## 4. Currently available treatments for LRTIs and their limitations

Antibiotics are the mainstay treatment for bacterial lung infection and are commonly selected considering the severity of the infections; in addition, the patient's age and other complications are considered while prescribing.^23^ Currently, there are two types of antibiotic treatment available for LRTIs; the first one is antibiotics for community acquired pneumonia (CAP) and another one is for hospital acquired pneumonia (HAP). CAP is treated with several antibiotics including fluoroquinolone, nemonoxacin, zabofloxacin, delafloxacin, tetracycline, macrolide, cephalosporin, pleuromutilin, and streptogramins.^24^ However, most recent investigation shows that amoxicillin and clarithromycin are the most commonly prescribed antibiotics from these drug groups for acute LRTIs.^25^ In contrast, HAP treatment is limited to some specific groups of antibiotics including dihydrofolate-reductase inhibitors, lipoglycopeptides and oxazolidinone.^24^ Most of these antibiotics target the bacterial DNA gyrase enzyme and show activity by inhibiting DNA replication,^26^ thus reducing mutant selection and toxic side effects.^27^ Both oral and parenteral therapies are available for LRTIs with dosage requirement for 8–12 hours for 7–10 days with variable doses from 400–875 mg according to the severity of the patient's symptoms.^28^ However, evidence shows that the antibiotics administered orally do not always help patients to recover baseline lung function, owing to the inaccurate drug concentration to the target site leading to the bacterial resistance against the antibiotics.^29^ In addition, these currently available oral antibiotic therapies are administered with high doses and frequent dosing.^30^ Increasing doses of drugs cannot overcome the problem; rather, it contributes to microbial resistance.^31^ Researchers prioritize their attention on the selection of the most effective and the least toxic antibiotics,^32^ but there is still a need to find better delivery methods to minimize dose related adverse effects. Health-care professionals specialised in antimicrobial stewardship still seek help to determine appropriate antibiotic choice, dose and minimizing resistance.^33^

LRTIs have huge impact on the global economy as a large amount of cost is associated in the management of LRTIs.^34^ Treatment for LRTIs causes enormous economic burden including direct and indirect financial losses; the estimated financial cost for antibiotic treatment associated with this disease is more than US$109 million each year globally.^35^ Because of the limitations of the currently available treatment systems, it is necessary to develop a better management system for the successful treatment of LRTIs at a very low dose of drugs with reduced costs. Pulmonary drug delivery could be utilized to substitute the currently available treatment system for LRTIs as it provides rapid onset of action, better therapeutic effects at a very low dose owing to the large surface area for drug absorption and is user friendly.^36^ In addition, inhaled antibiotics are administered in low doses and do not show the potential drug-related toxicity risks.^37^ Very limited studies have been done on pulmonary drug delivery for the effective treatment of LRTIs. Therefore, extensive studies are warranted to extend the application of pulmonary drug delivery for the management of LRTIs.

## 5. Pulmonary drug delivery system

Pulmonary delivery of drugs is a well-established drug delivery technology for the management of asthma, COPD and CF. It has been reported that pulmonary drug delivery is effective in treating the respiratory tracts.^38^ It has attracted significant attention in the last three decades for both the local and systemic therapeutic outcomes by offering lungs an optimistic pathway of non-invasive drug delivery. Thus, inhaled medication has become an interesting alternative route of drug administration for managing diseases related to respiratory tracts *i.e.* asthma, COPD, cystic fibrosis and lung infections such as pneumonia.^39–43^ The pulmonary route of drug delivery using biotherapeutics and macromolecules (*e.g.* growth hormones, protein, vaccine, and gene therapy)^44–47^ has shown promising effects both in local and systemic illness. Currently, only one inhaled dry powder inhaler product (tobramycin, Tobi Podhaler) is available for the treatment of *P. aeruginosa* infection associated with CF. It has shown greater efficiency compared to that of other routes by achieving the bioavailability and efficacy at a very low dose. However, successful delivery of drugs through the pulmonary route is governed by three factors, drug formulations, suitable drug delivery devices and the patient's inspiratory force,^48^ which are detailed in the following sub-sections.

### 5.1. Drug formulations

Drug formulations are considered as the most important factor for the successful delivery of drugs into the infected airways. Inhaled formulations of the micronized drugs (<5 μm) include dissolved/suspended drug particles in a suitable solvent (suspensions or emulsions) or powder formulation mixed with a large carrier (lactose) with improved flow properties for efficient aerosolization. Micronized or nanosized dry powders offer maximum potentials to deliver the formulated drug particles into the lower parts of the lungs as they can avoid the defense mechanisms of the lungs, being very negligible in size. The distribution of the inhaled drug particles in the lung depends on the characteristics of the inhaled particles, such as drug particle diameter, mass, shape, density and hygroscopicity,^49^ the physiology of the respiratory tract, and breathing patterns of the patients.^50^ Increased flow properties of the formulated drugs ensure that a desirable amount of drugs could be deposited into the targeted sites.^51^ To increase the flow properties, the formulation of drugs can be engineered by several methods including mixing with dispersibility enhancers, preparing particles by spray drying, freeze-drying and supercritical fluids to get controlled properties including size and shape. In a study, Islam *et al.*^52^ investigated the effect of fine particles of lactose on the aerosolization of salmeterol xinafoate from the dry powder inhaler formulation, and fine lactose played a key role in increasing the drug dispersion; however, the size of the large carriers had a limited impact on the drug aerosolization.^53^ Islam *et al.*^54^ continued their studies to determine the drug carrier force by using atomic force microscopy (AFM), which showed a significant impact of the carrier particle surface roughness on the dispersion of the adhered drug. However, these studies were extensively based on lactose carriers and could not provide a clear understanding of the effects of other carriers. Excipients could be used as additives with the formulated drug to get better flow properties.^55^ Only a limited number of excipients are available to increase the flow properties of the formulated drug particles, especially for pulmonary routes.^56^ The formulated drug particle size should be less than 5 μm, which is highly cohesive and cause poor flow properties which results in poor dispersion and dose variation; therefore, the desired amount of drug could not reach into the deep lungs.^57^ So far, various excipients have been used to improve the efficiency of pulmonary drug delivery including polymers, surfactants, sugars, amino acids, lipids and absorption enhancers.^56^ Evidence shows that carriers or excipients with active pharmaceutical ingredients used in pulmonary formulations could provide better therapeutic effects than the conventional drug delivery systems.^58^ Lactose is the most preferred excipient for pulmonary drug formulations which has been extensively used to increase the flow properties of poorly dispersible drug particles.^59^ However, more needs to be done to increase the flowability of the formulated drugs and their excipients.

### 5.2. Drug delivery devices

Three principal devices are available for pulmonary drug delivery: nebulizers, metered dose inhalers and dry powder inhalers.

#### 5.2.1. Nebulizers

A nebulizer is a polyvalent device which works as a passive administrator of therapeutic ingredients to the patients.^60^ Nebulizers aerosolize drug solutions or suspensions and deliver the aerosolized drug into the lungs by a spacer.^61^ They are mainly used for emergency purposes^62^ and need support personnel to administer the drug. There are two types of nebulizers on the basis of their mechanisms: (a) air jet nebulizers which use compressed air to aerosolize the formulations and (b) ultrasonic nebulizers which use vibrations of a piezoelectric crystal to aerosolize the formulations.^63^ Several nebulizers are currently available in the market with increased portability, convenience and energy efficiency, especially the mesh nebulizers.^64^ However, they require large volume/mass as most of the drugs are either retained inside the nebulizer or lost in the environment. It has been reported that only ten percent of the applied dose is deposited into the infected lungs.^65^ Thus, it is only suitable for hospital use with a high-dose drug setting as an inhaler device for the patients.

#### 5.2.2. Metered dose inhalers

Metered dose inhalers (MDIs) are a pressurized system in which drugs are dissolved or dispersed in a propellant. It consists of a pressurized canister of medicine in a plastic case with a mouthpiece. A holding chamber consists of a plastic tube with a mouthpiece, a valve to control mist delivery and a soft sealed end to hold the MDI. The holding chamber assists the delivery of medicine to the lungs.^66^ Although it is portable and convenient, its use is limited due to being expensive and flammable. Only 10–30% of the total drug sprayed from MDIs reaches the lungs,^67^ and the rest of the dose is deposited in the oropharynx.^68^ Pressurized metered dose inhalers (pMDIs) also cannot overcome this limitation by providing only 20–50% deposition in the lungs.^69^ In addition, metered dose inhalers are not environment friendly as they produce chlorofluorocarbons (CFCs) which accumulate in the stratospheric layer of the Earth's atmosphere and destroy the protective ozone layer.^70^

#### 5.2.3. Dry powder inhalers

Dry powder inhalers (DPIs) are one of the most commonly used lung delivery devices which carry drugs as loose agglomerates of micronized drug particles or carrier-based (lactose as a common carrier) interactive mixtures with micronized drug particles adhered onto the surface of the large carriers.^71^ It is one of the best drug delivery devices for pulmonary delivery as it is highly portable and physiochemically stable as drugs are kept in the solid state in the devices, particularly for proteins and peptides.^72^ In addition, a DPI is more suitable as it is being delivered in powder forms which is capable of carrying poorly water-soluble drugs, protein based formulations and peptides.^73^ DPIs are formulated using inhalable micron sized drugs (<5 μm) with a combination of large coarse carriers (90–150 μm) or agglomerates of drug particles with controlled flow properties. An appropriate delivery device is required to deliver the formulated drugs into the deep lungs while the inhaler device usually functions using the patient's inspiratory force.^74^ Successful drug administration by the DPI is governed by 3 factors: the interparticulate forces among the formulated powder, the dispersion forces generated during inhalation and the deposition forces in the human respiratory tract.^75^ However, Schiavone *et al.*^57^ demonstrated that the micron-sized drug particles in DPI formulations showed poor dispersion due to cohesive forces among the drug particles. They concluded that advanced particle engineering could be an option to improve the flow properties of the inhalable formulations. Coarse carriers commonly use lactose, which breaks these cohesive drug agglomerates and forms weaker adhesions between the carrier and the micronized particles.^76^ Investigations showed that the carrier size, shape, charge, surface morphology have a great impact on the aerosolization of the formulated drug particles and drug release pattern from the carriers in a DPI formulations.^77^ However, evidence also shows that the molecular weight of the drugs and balance of hydrophilic and lipophilic properties among the formulated powders also play roles in the aerosolization properties of the DPI formulations.

### 5.3. Patient's inspiratory force

Another important factor for successful drug deposition into the infected airways is the patient's inspiratory force. Upon inhalation from the delivery devices, the formulated drugs are introduced, and the inspiration force aerosolizes the powder bed by shear and turbulence^60^ and particles enter into the patient's airway. After inhalation, the particle size of the inhaled drug determines the deposition of drug in different regions of the lungs. Generally, particles need to be less than 2 μm for their successful deposition into the deep lungs.^78^ A slow breathing pattern is incapable of carrying the drugs into the infected areas, especially into the deep lungs; instead, they are deposited into the small airways, bronchioles and alveolar regions with suitable size and mass.^79^ Drug insufflation, carrier-based DPI formulation drugs are detached from the surface of the large carrier particles and deposited into the deep lungs while the large carriers impact the oropharynx and are cleared.^80^

## 6. Challenges of drug delivery by the pulmonary route in the infected lungs

The challenges of delivering drugs within the targeted area include minimizing its degradability, increasing its bioavailability, and furthermore reducing its cellular toxicity. In a study, Douafer *et al.*^63^ described various devices and techniques which have been used to overcome the challenges and achieve proper therapeutic effects of the administered drugs. However, lung physiology needs to be considered as a controlling factor to utilize any devices or techniques for lung drug delivery. Lung delivery of drugs also faces challenges including multiple filtrations through the respiratory tract, an innate immunological response and rapid exhalation from the lungs.^81^ However, three mechanisms such as impaction, sedimentation and diffusion have been demonstrated to overcome these challenges.^82^ Thus, the challenges for successful drug delivery into the infected lungs could be discussed in two sections: drug deposition in the infected airways and bacterial colonies/biofilms.

### 6.1. Drug deposition in the infected airways

Inhaled particles <5 μm in size through the pulmonary route normally reach the deep lungs in healthy patients. However, the lung's natural defense mechanism also creates a barrier for the inhaled particles as foreign particles. There is a thick mucus protective layer in the upper airways (from the windpipe to the tertiary bronchi) that trap and clear foreign particles by either coughing or swallowing.^83,84^ The clearance of particles from this region is also governed by the number of cilia and the ciliary beat frequency, as well as the quality and quantity of mucus.^85^ Bissonnette *et al.*^86^ reported that the alveolar region serves as barriers for the transportation of molecules in the deeper areas of the lungs, *e.g.* a barrier lining of a variety of proteins and lipids, the compact junction present in the epithelial cell, alveolar macrophages, *etc.* Additionally, lung infection causes mucosal swelling of airway which results in narrowing of the cross-sectional diameter of the airways than that of a healthy lung. Excessive mucus production and deposition in infected lungs cause more turbulence of the inhaled air.^87^ Consequently, inhalable particles (<5 μm) deposit on the central airway mucosa instead of reaching the deep lungs. Thus, infected airways create more barriers for the inhaled drugs to reach the targeted site. Besides, they can cause local detrimental effects including bronchospasm and coughing due to deposition in the upper respiratory tracts, which may uplift patient compliance.^88^ Nanotechnology and particle engineering techniques were found to be efficient in overcoming this barrier of infected lungs for pulmonary drug deposition,^89–91^ and drug nanoparticles with a diameter of 1–200 nm can avoid entrapment in the upper airway and it can reach the alveolar sacs for absorption and rapid onset of action.^92^ Nanotechnology was also found advantageous for the objective of improving drug solubility, dissolution profiles and pharmacokinetic profiles and reducing the premature mucociliary clearance of hydrophobic drugs.^93^

### 6.2. Bacterial colonies/biofilms

Bacterial colonies inside the infected lungs are immersed in a dense immobilized mucus layer which acts as the solid barrier to the antibiotic exposure.^94^ Polynomic contents of the mucus (*e.g.* mucin, actin, and DNA) physically bind to antibiotic molecules by both electrostatic and hydrophobic interactions^95^ and hence prevent antibiotic molecules from reaching the bacterial colonies. Besides, the obstructed circulation of the antibiotic particles due to the presence of the mucus barrier makes them highly vulnerable to the lung phagocytic clearance, causing their short retention time in the lung.^96,97^ However, DPI nanoparticles (<200 nm) are found effective in mucus penetration due to their smaller size.^92^ Even they can avoid unfavourable mucociliary clearance and phagocytic clearance^98^ by remaining in the lung lining fluid until dissolution^99^ or translocation by the epithelial cells.^100^ It is desirable that nanotechnology could overcome bacterial colony forming challenges easily using micron sized DPI formulations, as the aim of antibiotic DPIs for lung infection treatment is to exert a bactericidal effect and better management of the disease.

## 7. Polymer–drug conjugated nanoparticles for pulmonary delivery

**Fig. 2:**
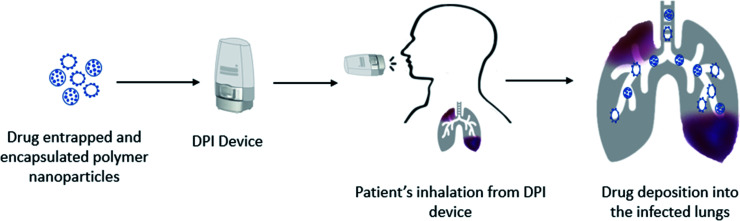
Schematic diagram of pulmonary drug delivery from DPI formulations. The formulation consists of drug entrapped and encapsulated in polymer nanoparticles. Nanoparticles are aerosolized using a DPI device and patient's inspiratory force and deposited into the infected lung.

Drug formulations for pulmonary delivery can be developed as a controlled release (CR) profile which ensures the release of the formulated drugs gradually and predictably over extended periods, thus maintaining a constant plasma concentration. The CR provides the formulated drugs greater effectiveness to treat persistent conditions as the medication is given consistently. It adds benefit over immediate drug release by reducing side effects while improving patient compliance as doses are in a simplified schedule. CR profiles are dependent on the formulation of drugs with specified particle size, shape, and surface properties. Polymers have been used extensively as carriers to investigate their CR patterns. Both synthetic and natural polymers showed encouraging CR patterns including, poly(lactic-*co*-glycolic acid) (PLGA), poly(ε-caprolactone) (PCL), polyvinyl alcohol (PVA), poly(lactic acid) (PLA), poly(ether-anhydride), chitosan, sodium hyaluronate and albumin.^101–103^ Particle engineering of biodegradable polymers as carriers has shown protection to the encapsulated drugs and stable drug delivery from the formulations and efficient transportation to the targeted sites. However, micron size drug particles with suitable carriers still face challenges to achieve CR, thus requiring more suitable nano-sized drug formulations. Drug-encapsulating polymer nanoparticles could be an alternative tool to overcome this limitation with the desired CR profile. Drug loaded polymer micro- or nano-particles are the most recent advanced technology of drug formulations towards pulmonary drug delivery. Thus, the selection of a proper drug delivery technique is a critical issue to avoid the defense system of the lungs which have a strict clearance mechanism and multiple barriers against foreign insertion.^104,105^ Nanotechnology has a promising scope to utilize it in pulmonary drug delivery to avoid first pass metabolism and rapid absorption because of its small size, and physical and chemical characteristics including large surface area.^106,107^ Nanoparticles in the form of DPI formulations (Fig. 2) are considered as the most feasible delivery method of drugs embedded within a carrier rather than the direct pulmonary administration of nanoparticles.^108^ Nanoparticles showed agglomerates and compromised deposition behaviour of particles because of the high surface energy, while appropriate excipients in DPI formulations showed promising deposition and deagglomeration behaviour.^109^ Drug loaded polymeric nanoparticles can carry the drug into the infected lungs either by loading the drug molecules on the surface of the polymers or encapsulating the drug molecules within its matrix.^110^ Interestingly, after deposition into the infected airways or into the deep lungs where a humid environment exists, the polymer matrix dissolves and releases the nanoparticulate drug molecules.^111^ The polymer encapsulated siRNA targeting lung cancer showed less toxic effects within the cells because of the preferential accumulation of the drug in the target cells and also found reduced toxic effects of celecoxib-loaded PLGA nanoparticles on lung cancers.^112,113^ Thus, the engineered polymeric nanoparticles are capable of carrying the encapsulated drugs into the target areas and make them suitable to provide better therapeutic effects at a very low dose and minimize dose associated toxic effects. Polymeric nanoparticles are designed for targeted delivery,^114^ and sustained delivery^58,115,116^ of drugs in the deep lungs^109^ by DPIs. Wu *et al.*^117^ demonstrated that DPI formulations of polymeric cyclosporine drug nanoparticles (1–100 nm) are effective in producing highly aerosolized particles. The surface charge of modified polymer drug nanoparticles was found to improve antibiotic delivery in the infected lungs.^118^ However, this study could not provide the cytotoxic effects of the synthetic lung surfactant-mimic phospholipid, 1,2-dipalmitoyl-*sn-glycero*-3-phosphocholine and 1,2-dipalmitoyl-*sn-glycero*-3-(phosphor-*rac*-1-glycerol) sodium salt. Salvati *et al.*^119^ studied the surface properties of the polymeric drug nanoparticle (cationic charged/neutral) and found it to adhere to the mucus layer of the lungs and exert sustained drug release. In another study, Huang *et al.*^120^ found that inhaled nanoparticles were effective in the alteration of drug interactions with target cells, owing to the preferential accumulation. Thus, polymer–drug conjugated nanoparticles are suitable to be utilized in pulmonary delivery with precise delivery for both local and systemic effects.

## 8. Polymer based pulmonary delivery

Polymers are considered as attractive carriers of drugs to the lung. They offer easy encapsulation strategies for drugs within them in various forms including nanoparticles, microparticles and nano-embedded microparticles.^111,121^ In addition, polymers could slow the systemic absorption of conjugated delivery, thus increasing the drug sustainability within the lung. This is helpful for the treatment of lung related illness, but the drug needs to be deposited within the lung for a longer period rather than instant absorption.^122^ Both natural and synthetic polymers have been extensively studied for pulmonary drug delivery as carriers or excipients for DPIs,^123^ facilitating the aerodynamic properties, inhibiting particle aggregation, and thus increasing particle dispersion and deposition.^124^ However, synthetic polymers showed more effective drug release profiles in a sustained way over the natural polymers.^122^ The choice of suitable polymer carriers for drug encapsulation depends on their biodegradability, encapsulation efficacy, drug release pattern and easy formulation properties.^125^

### 8.1. Natural polymers for pulmonary delivery

Among natural polymers, chitosan and alginate have extensively been studied for their biocompatibility, biodegradability, and low-toxicity behaviour for lung drug delivery. Rohani *et al.*^152^ demonstrated the enhanced fine particle fraction (FPF) (46–81%) of the spray-dried chitosan encapsulated insulin powder formulation. They suggested that excipients including mannitol, sodium alginate and sodium citrate helped increase the flow properties by changing the surface properties of the formulated powder. Al-Qadi *et al.*^126,127^ showed the improved aerosolization properties of the protein-loaded chitosan nanoparticles over other formulations. The improved absorption of the nanoparticles occurred due to the interaction between the cationic chitosan with the target cells. For example, protein encapsulated chitosan showed improved systemic absorption with prolonged drug release upon lung delivery.^128^ Thus, chitosan has been positioned as a promising carrier for pulmonary delivery of various drugs. Antibiotic-encapsulating chitosan microparticles and nanoparticles showed promising effects to improve drug deposition, dispersibility, tissue uptake, and modified surface characteristics for better aerosolization.^91^ In addition, anticancer, anti-asthma antifungal and antihypertensive drugs were also investigated for pulmonary delivery. Promising characteristics of chitosan to improve drug delivery of pulmonary drugs encouraged its use in other delivery routes, as most recently modified chitosan in nanobioconjugate photosensitive nanocarriers was studied to determine its compatibility with other biological carriers including azobenzene molecules, thus showing improved therapeutic effects in cardiac delivery.^129^ However, safety issues related to chitosan and its derivatives in lung delivery makes it a less preferable choice in recent studies.^91^

Another natural polymer which has been extensively investigated for pulmonary drug delivery is alginate due to its low cost, ease of preparation, mucoadhesiveness, biocompatibility and nontoxic characteristics.^130,131^ However, alginate loaded micro- or nano-particles also limits its use due to its rapid drug release characteristics.^132^ Although various studies have been done to improve its drug release pattern, still a lot needs to be done to determine the alginate improved drug release pattern in combination with other polymer carriers.^130^Table 1 shows some of the natural polymers which have been used as carriers in pulmonary drug delivery.

**Table 1:** Natural polymer based pulmonary delivery^a^

Polymer	Encapsulated molecules	Main findings	Ref.
Chitosan	Isoniazid	Prolonged drug release and improved aerosolization	136
Crosslinked chitosan	Levofloxacin	Improved aerosolization	137
Chitosan	Ciprofloxacin	Improved aerosolization and therapeutic effects	138
Chitosan	Isoniazid and rifampicin	Improved bioavailability and cellular uptake	139
Chitosomes (chitosan–xanthan gum)	Liposomes	Improved aerosolization	140
Chitosan	Ethambutol dihydrochloride	Improved bioavailability and cellular uptake	141
Chitosan	Dapsone	Prolonged drug release and improved aerosolization	142
Chitosan	Ofloxacin	Improved aerosolization and cellular uptake	143
Chitosan	Moxifloxacin	Improved cellular uptake	144
Chitosan	Rifampicin and rifabutin	Prolonged drug release; improved aerosolization and bioavailability	145
Chitosan/fucoidan	Gentamicin	Prolonged drug release and improved bioavailability	120
Chitosan	Vancomycin	Improved bioavailability and cellular uptake	146
Alginate	Paclitaxel	Prolonged drug release and improved bioavailability	147
Alginate/chitosan	Tobramycin	Improved bioavailability and cellular uptake	148
Alginate	Isoniazid, rifampicin, and pyrazinamide	Prolonged drug release	149
Alginate/PLGA	Amikacin, ciprofloxacin and polymyxin	Improved aerosolization	150
Alginate/chitosan	PR8 influenza virus	Improved bioavailability	151
Chitosan	Insulin	Improved dispersibility	152
Alginate	Poloxamer	Prolonged drug release	153

a Abbreviation: PLGA – poly(lactic-*co*-glycolic acid).

### 8.2. Synthetic polymers for pulmonary delivery

Synthetic polymers showed promising effects over natural polymers for being easily synthesizable and cost effective. Various synthetic polymers have shown promising effects, being biocompatible and versatile including polyanhydrides, poly(lactic acid) (PLA) and poly(lactic-*co*-glycolic-acid) (PLGA).^133^Table 2 shows some of the synthetic polymers which have been used as carriers in pulmonary drug delivery. Synthetic polymer nanoparticles showed enormously positive characteristics over microparticles; thus, research nowadays is extensively focused on nanoparticle based pulmonary delivery.^134^ Recent findings from aerosol delivery of polymer-conjugated drug particles encouraged Zhang *et al.*^135^ to study it in neurodegenerative diseases using baicalein loaded poly(ethylene glycol)-*block*-poly(d, l-lactide) (PEG–PLA) conjugated particles, and the findings were promising against oxidative stress and inflammation.

**Table 2:** Synthetic polymer based pulmonary delivery^a^

Polymer	Encapsulated molecules	Main findings	Ref.
PLA/PLGA	Hepatitis B vaccine	Prolonged drug release and improved bioavailability	154
PLGA	Rifampicin, isoniazid and pyrazinamide	Improved bioavailability	155
PLGA	Rifampicin	Prolonged drug release	156
PLGA	Pirfenidone	Prolonged drug release and improved bioavailability	157
PLGA	Voriconazole	Prolonged drug release and improved aerosolization	158
PLGA/chitosan	Calcitonin (peptide)	Prolonged drug release	159
PLGA/chitosan	Exendin-4	Prolonged drug release	160
PLGA/polyethyleneimine	DNA vaccine	Increased therapeutic effects	161
PLGA/polyethyleneimine	siRNA	Increased therapeutic effects	112
PLGA/PEG	Velcade	Increased therapeutic effects	162
PEG	Plasmid DNA	Improved cellular uptake	163
PLGA/Fe_3_O_4_	Quercetin	Increased therapeutic effects	164
PVA/PLGA	siRNA	Improved bioavailability	165
PVA/PLGA	Salbutamol	Prolonged drug release and improved therapeutic effects	166

a Abbreviation: PLA – polylactic acid; PLGA – poly(lactic-*co*-glycolic acid); PVA – poly(vinyl alcohol); PEG – poly(ethylene glycol); PCL – polycaprolactone.

## 9. Antibiotics used in DPI formulations for pulmonary delivery

Antibiotics are considered as the first line treatment for lung related diseases, especially for LRTIs. As discussed earlier, currently available antibiotic therapies are in high doses and need frequent administration, thus increasing cellular toxicity and antimicrobial resistance. To overcome the limitations of the currently available antibiotic formulations, research has been focused on the nanoparticulate DPI formulations of these antibiotics. Table 3 shows some of the antibiotics which have been sufficiently formulated into aerosolized nanoparticles for pulmonary delivery. Both natural and synthetic polymers were studied extensively to improve the therapeutic effects of the formulated antibiotic-loaded nanoparticles. Besides, in many cases, excipients were used to increase the powder flowability of the prepared formulations while formulating aerosol particles for lung delivery.^167,168^ However, still their effectiveness needs to be tested in a standard lab and human trials to determine their complete safety profile.

**Table 3:** Inhalable antibiotic/drug nanosized DPI formulations^a^

Antibiotics/drugs	Carrier	Formulation technique	Excipients	Findings	Ref.
Vancomycin and clarithromycin	DPPC	Spray drying	—	Improved aerosolization	178
Tobramycin and azithromycin	Organic solution	Spray drying	—	Improved aerosolization	89
Rifampicin	PLGA	Solvent evaporation/ spray drying	PVA and l-leucine	Prolonged drug release	156
Moxifloxacin and ofloxacin	DPPC	Spray drying	—	Improved aerosolization	179
Isoniazid	Chitosan/TPP	Spray drying	Lactose, mannitol, maltodextrin, and leucine, glycerine	Improved aerosolization	136
Isoniazid and rifampicin	HPMC	Precipitation	Mannitol, leucine, and Tween 80	Improved aerosolization and prolonged drug release	180
Tobramycin	PLGA/Chitosan	Emulsion/solvent evaporation	—	Improved aerosolization	181
Aspirin and salbutamol	Polyacrylate	Spray drying	Tween 20	Prolonged drug release	182
Levofloxacin	PCL/PVA	Emulsion/solvent evaporation	d-Mannitol and l-leucine	Improved aerosolization	183
Ciprofloxacin and levofloxacin	PLGA/PCL	Emulsion/solvent evaporation	—	Improved drug penetration	92
Levofloxacin	PLGA/Lecithin (lipid)	Spray drying/Spray freeze drying	d-Mannitol, l-leucine, and PVA	Improved aerosolization	184
Rifampicin	PLGA/PVA	Emulsion/solvent evaporation	Lactose	Improved aerosolization	185
Clarithromycin	PLGA/PVA	Emulsion/solvent evaporation	Mannitol, l-leucine, and lactose	Improved aerosolization	186
Ciprofloxacin	Polyacrylate	Spray drying	l-Leucine and lactose	Improved aerosolization and prolonged drug release	187
Levofloxacin	PCL	Spray drying	Pluronic F-68, d-mannitol, lactose, and l-leucine	Improved aerosolization	188
Levofloxacin	PCL	Emulsion/solvent evaporation	l-Leucine, PVA, α-lactose monohydrates, and d-mannitol	Improved aerosolization	189
Rifampicin	Chitosan	Ionotropic gelation	—	Prolonged drug release and improved cellular uptake	190
Isoniazid, rifampicin, and pyrazinamide	Alginate	Cation-induced gelification	Chitosan	Prolonged drug release	191
Rifampicin, isoniazid, and pyrazinamide	PLGA	Emulsion/solvent evaporation	—	Improved bioavailability	155
Pirfenidole	PLGA	Emulsion/solvent evaporation	PVA	Improved bioavailability	157
Salbutamol	Poly(vinyl sulfonate-*co*-vinyl alcohol)-*g*-PLGA	Modified solvent evaporation	—	Prolonged drug release	166
Ibuprofen	PEG–PLGA	Emulsion/solvent evaporation	—	Improved mucus penetration	192
Ethionamide	PLGA	Emulsion/solvent evaporation	Lactose	Prolonged drug release	193
Tobramycin	PEG–PLGA	Emulsion/solvent evaporation	—	Improved bioavailability	194
Tobramycin	PLGA	Spray drying	—	Improved cellular uptake	195
Rifampicin	PLGA	Spray drying	—	Prolonged drug release and improved cellular uptake	196
Rifampicin	PLGA	Emulsion/solvent evaporation	l(+)-Arginine and l-leucine	Improved aerosolization	197
Ciprofloxacin	PLGA	Nanoprecipitation	Pluronic F-68	Improved aerosolization	198
Ciprofloxacin	PLGA	Nanoprecipitation	Pluronic F-68	Improved aerosolization	199

a Abbreviation: DPPC – 1,2-dipalmitoyl-*sn-glycero*-3-phosphocholine; PLGA – poly(lactic-*co*-glycolic acid); PVA – poly(vinyl alcohol); TPP – tripolyphosphate; HPMC – hydroxypropyl methylcellulose; PCL – polycaprolactone; PEG – poly(ethylene glycol).

## 10. Preparation techniques for drug-encapsulating polymer nanoparticles

Drug conjugated polymer nanoparticles for pulmonary delivery have some special features that should be considered during formulation and design. Several methods have been developed to control the particle size distribution, increase stability, and improve the CR profile and targeted delivery with enhanced bioavailability. So far, spray drying has been the most popular technique for preparing inhalable powders. Freeze-drying or lyophilization has also been investigated as a method to produce a solid dry powder that could be administered through lung delivery or after rehydration in the appropriate buffer. The selection of methods depends on the characteristics of the polymers, solubility of the drugs and stability of the formulated powder nanoparticles. The following methods have mostly been used for polymer-based drug nanoparticle formulations for lung delivery.

### 10.1. Double emulsion/solvent evaporation method

Solvent evaporation is appropriate for encapsulating both hydrophilic and lipophilic drugs with high efficiency.^169^ This method is performed in a water-in-oil (w/o) emulsion of the polymer and is being prepared using an appropriate surfactant. The stable emulsion of the polymer is cross-linked by an appropriate cross-linking agent such as glutaraldehyde to harden the droplets. The polymer nanoparticles are acquired by evaporation of the oil phase. The size of the prepared nanoparticles depend on the extent of the cross-linking agent, speed of stirring and aqueous droplet size; thus, formulated nanoparticles have been shown to demonstrate enhanced mucoadhesiveness or cellular absorption upon drug delivery.^170^ However, drawbacks of this method include a tedious procedure, harsh cross-linking agents and difficulty to wash away the cross-linking agent.^171^

### 10.2. Spray-drying method

Spray-drying is a process where mechanical high energy input is avoided. Therefore, this technique is appropriate for thermolabile materials and macromolecules such as peptides and proteins. Polymers with/without drugs are primarily dissolved or dispersed in an organic solvent (*e.g.* acetic acid/dichloromethane), and then a suitable cross-linking agent is added to this formulation. An outflux of hot air atomises the solution into small droplets of free-flowing nanoparticulate powders. Particle size depends upon the size of the nozzle, spray flow rate, atomisation pressure and inlet air temperature, and extent of cross-linking. The hot air can degrade the heat-liable substance.^172^

### 10.3. Antisolvent precipitation/salting-out method

The anti-solvent method is widely used in pharmaceuticals to produce very fine particles with specific particle surface morphology and physical state. Acetone is commonly chosen as the water-miscible organic solvent in this method as acetone is pharmaceutically acceptable in terms of toxicity.^173^ Poly(vinyl alcohol) is added in the organic solvent (acetone), which contains the polymer in it and highly concentrated salt solution. High salt concentration in the aqueous phase prevents the mixing of acetone with pure water despite being miscible. After emulsification, the addition of water in a sufficient quantity causes acetone to diffuse into the aqueous phase, resulting in the formation of nanoparticles.^174^

### 10.4. Ionotropic gelation (polyelectrolyte complexation: TPP method)

This method encapsulates a drug by the interaction of an ionic polymer with oppositely charged ion to initiate cross linking.^175^ To prepare nanoparticles, a polymer (*e.g.* chitosan) is dissolved in an aqueous solution (*e.g.* cation of chitosan); then a poly anionic tripolyphosphate (TPP) is added dropwise to the solution under constant stirring. As a result of complexation between oppositely charged species, the polymer undergoes ionic gelation and precipitates to form spherical particles. The resulting polymer particle suspension needs to be centrifuged and dried subsequently. This method is environment friendly as water can be used as the solvent. Prepared nanoparticles have been demonstrated to enhance mucoadhesiveness or cellular absorption upon pulmonary delivery.^176^ The self-assembly method of drug-encapsulating polymer nanoparticles is a recent addition in the field of nanotechnology. Hydrogen bonding may be used to form nanoparticles between the neutral polymers and tannic acid. This method was used to prepare doxorubicin-encapsulating poly(2-oxazoline) nanoparticles for cancer treatment.^177^ This technique could be used for drug-encapsulating polymer nanoparticles for pulmonary delivery as well.

## 11. Conclusion and future directions

This article provides a comprehensive critical review of the current status of LRTIs, the limitation of the current management system and the development of better delivery technology. LRTIs are still considered a life-threatening disease, and the currently available treatment system has dose-related adverse effects. Besides, the direct and indirect cost associated with the management system of the LRTIs has a huge economic impact on the world economy. Thus, the establishment of a cost-effective management system for LRTIs is on the priority list of the scientists. Inhaled antibiotics have drawn the attention of researchers as an efficient management system to overcome the economic burden. Research in developing drug-encapsulating polymer nanoparticles for pulmonary delivery is progressing and has achieved considerable success so far. Nanoparticulate drugs can diffuse through the mucus layer of the infected lungs, release drugs in the cells and produce better therapeutic action at a low dose. However, some commonly used polymers such as chitosan are raising concerns on their biodegradability characteristics. Therefore, *in vivo* optimization is required to determine the safety of the polymer nanoparticles for lung cells. Given the potential for improved treatment by delivering drugs into the deep lungs, further research is essential to develop efficient inhaled antibiotic formulations for the proper management of LRTIs in the future.

## Author contributions

Mohammad Zaidur Rahman Sabuj: conceptualization, design, original draft preparation, editing and revision of manuscript. Nazrul Islam: supervision, conceptualization, review & editing.

## Conflicts of interest

The authors declare no conflict of interest.

## Supplementary Material
